# Considerations for post‐licensure group B streptococcus vaccine effectiveness studies

**DOI:** 10.1002/ijgo.14845

**Published:** 2023-05-11

**Authors:** Helen Skirrow, Dan Kajungu, Kirsty Le Doare, Tracey Chantler, Beate Kampmann

**Affiliations:** ^1^ Department of Primary Care and Public Health, School of Public Health Imperial College London London UK; ^2^ Makerere University Centre for Health and Population Research Iganga Uganda; ^3^ Department of Global Health Stellenbosch University Stellenbosch South Africa; ^4^ Centre for Paediatric and Neonatal Infection St. George's University of London London UK; ^5^ Makerere University, John's Hopkins University Kampala Uganda; ^6^ The Vaccine Centre, Faculty of Public Health & Policy London School of Hygiene & Tropical Medicine London UK; ^7^ Charite Centre for Global Health Berlin Germany

**Keywords:** group B streptococcal disease, pregnancy, vaccines

## Abstract

Post‐licensure studies of a Group B streptococcal vaccines for pregnant women in low and middle‐income countries will require investment in electronic health records.

1

A Group B Streptococcal (GBS) vaccine for pregnant women is likely to be licensed based on a correlate of protection.^1^ However, post‐licensure effectiveness studies will then be needed to evaluate the vaccine's impact on prevention of GBS disease among pregnant women and babies. Therefore, a priority now is to understand the preparedness of different health systems to deliver a GBS vaccine and undertake suitable post‐licensure surveillance studies.^2^

A stakeholder evaluation in Uganda and the United Kingdom (UK) involving maternal and child‐health practitioners, researchers and regulators aimed to describe operational strengths and gaps relevant to post‐licensure GBS vaccine studies. Convenience sampling was undertaken from IMPRINT's (Immunizing‐PRegnant‐Immunizing‐women‐and‐infants‐NeTwork) professional network with clinical, academic, and regulatory stakeholders approached by email and interviewed with their informed consent. In November and December 2020, nineteen interviews were conducted with midwifery (2), general‐practice (1), community‐health (1), pediatric (6), obstetric (2), microbiology (2) and public‐health regulatory (5) stakeholders: (10 from the UK and 9 from Uganda). The interviews focused on three areas relevant to post‐licensure vaccine studies; (i) existing data‐systems for the surveillance of pregnancy, birth, and maternal and infant outcomes; (ii) health‐system preparedness for delivering an additional pregnancy vaccine and (iii) wider stakeholder engagement for a GBS vaccine.

Stakeholders confirmed that in both the UK and Uganda established systems for offering vaccines to pregnant women existed which would facilitate the addition of a GBS vaccine. Awareness of GBS disease in Uganda was perceived to be much lower compared to the UK, but stakeholders in both countries felt that improved education and engagement about GBS disease was important for both pregnant women and healthcare workers prior to any vaccine introduction.^3^

The UK's strong existing data systems meant stakeholders considered it well placed for hosting post‐licensure GBS vaccine studies though enhancement of current mother and baby data‐linkage systems maybe required. In Uganda the lack of electronic health records outside of tertiary or research settings was seen as a major barrier to post‐licensure GBS studies (see Figure 1). This is likely to be reflective of other Low‐and‐Middle‐Income‐Countries situations. However, stakeholders also felt that further investment into existing research databases in Uganda (e.g. the Iganga‐Mayuge Health and Demographic Surveillance Site or PREPARE study sites) could result in suitable systems.^4^

**FIGURE 1:**
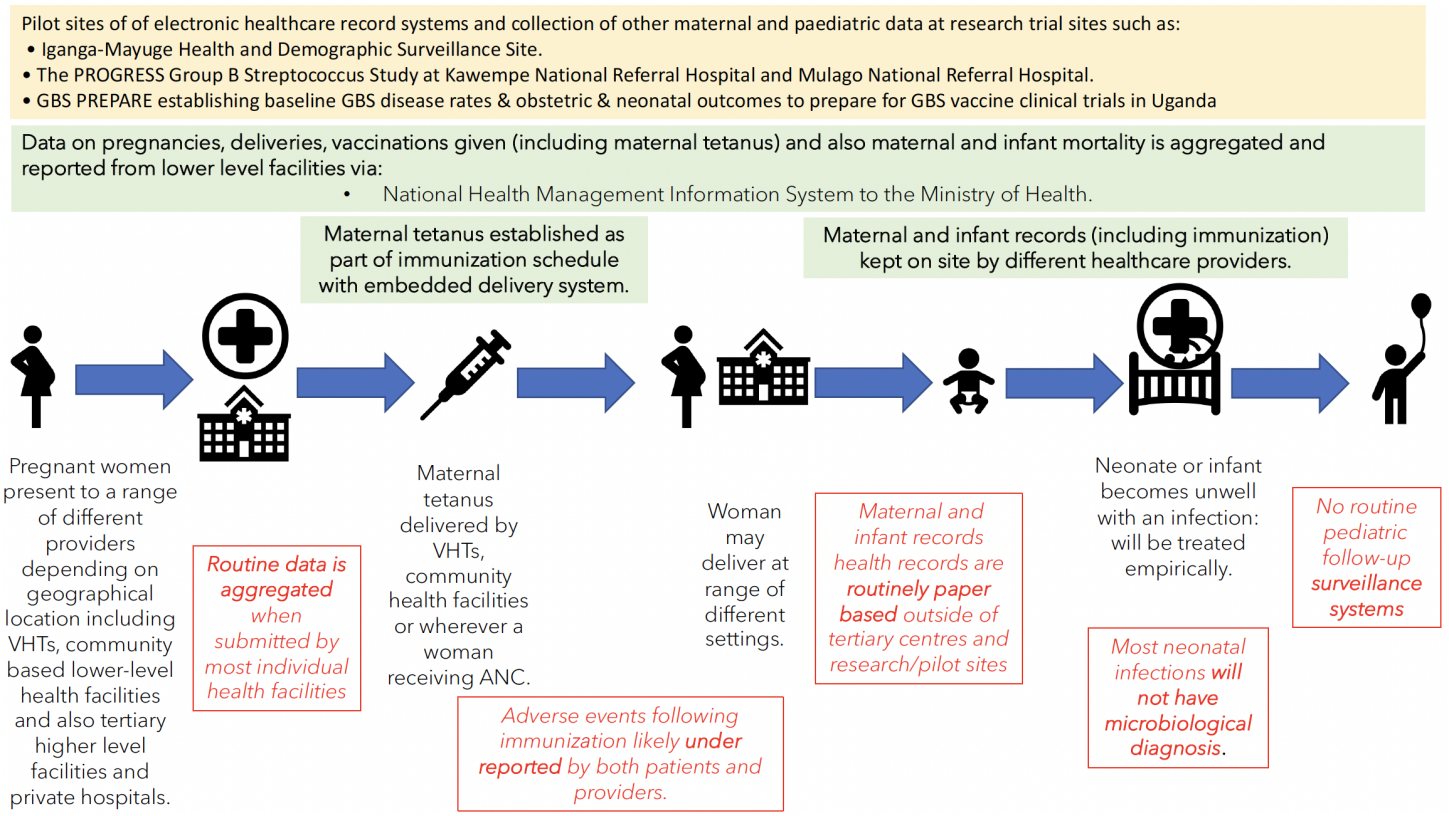
Existing data systems and healthcare system pathways for pregnant women and neonates in Uganda, indicating potential operational gaps. ANC, Antenatal Care; VHT, Village Health Teams.

In planning post‐licensure GBS effectiveness studies, each country's readiness needs careful consideration. To evaluate the impact of a future vaccine on GBS disease, timely investment is essential in low‐and‐middle‐income countries' electronic health systems.^2^

## AUTHOR CONTRIBUTIONS

HS contributed to design and carrying out the study, data curation, data analysis, writing original article draft and reviewing and editing. DK contributed to design and carrying out the study, and reviewing and editing article draft. KLD contributed to research design and carrying out the study, and reviewing and editing article draft. TC contributed to data analysis and reviewing and editing original article draft. BK conceptualized the idea for the study, contributed to design, analysis and carrying out the study, and reviewing and editing original article draft.

## FUNDING INFORMATION

HS, BK and DK were supported by funds from the Immunizing PRegnant women and INfants neTwork (IMPRINT) which is funded by the UK Research and Innovation‐Global Challenges Research Fund Networks in Vaccines Research and Development which was co‐funded by the Medical Research Council and Biotechnology and Biological Sciences Research Council.

BK is additionally funded by the MRC (MC_UP_A900/1122, MC_UP_A900/115).

HS is funded by National Institute for Health Research (NIHR), doctoral research fellowship award (NIHR300907).

KLD is funded by a UKRI Future Leaders Fellowship (MR/S016570/1) and the European and Developing Countries Clinical Trial Partnership (RIA2018V‐2304 ‐PREPARE). This article contains information about the PREPARE study, which is part of the EDCTP2 programme supported by the European Union (grant number RIA2018V‐2304‐PREPARE). The views and opinions of authors expressed herein do not necessarily state or reflect those of EDCTP.

TC is affiliated to the National Institute for Health Research Health Protection Research Unit (NIHR HPRU) in Vaccines and Immunization (NIHR200929) at London School of Hygiene and Tropical Medicine in partnership with UK Health Security Agency (UKHSA). TC is based at the London School of Hygiene & Tropical Medicine. The views expressed are those of the author(s) and not necessarily those of the NHS, the NIHR, the Department of Health or UKHSA.

## CONFLICT OF INTEREST STATEMENT

HS has undertaken a 6 month IMPRINT network funded fellowship during which time she was employed by Imperial College London (home institution) as a Clinical Research Fellow. This IMPRINT fellowship has involved HS undertaking shadowing at Pfizer UK vaccines trials team, however this has excluded any GBS or Covid‐19 vaccine trials shadowing. HS has not and will not, receive any money, stock options, payment in kind, travel expenses, food expenses, conference fees or any reimbursement from Pfizer or any associated companies. HS training agreement between the IMPRINT network, Imperial College London and Pfizer states that: ‘it is expressly understood and agreed that this Agreement does not intend and shall not be construed to create the relationship of agent, servant, employee, partnership, joint venture or association between the Home lnstitution or IMPRINT or its Fellows and Pfizer’.

## Data Availability

Research data are not shared.
